# A Rare Symptom

**Published:** 1882-12

**Authors:** C. Hering


### A RARE SYMPTOM.

Anxiety and impeded breathing has to leave the room and go into the air: Lauro.

"Anxiety and impeded breathing have, no doubt, been observed very often to drive one out-doors, but it is neither mentioned in the materia medica nor among the cures."—C. Hering.

